# Delta-like 4 inhibits choroidal neovascularization despite opposing effects on vascular endothelium and macrophages

**DOI:** 10.1007/s10456-012-9290-0

**Published:** 2012-08-07

**Authors:** Serge Camelo, William Raoul, Sophie Lavalette, Bertrand Calippe, Brunella Cristofaro, Olivier Levy, Marianne Houssier, Eric Sulpice, Laurent Jonet, Christophe Klein, Estelle Devevre, Gilles Thuret, Antonio Duarte, Anne Eichmann, Laurence Leconte, Xavier Guillonneau, Florian Sennlaub

**Affiliations:** 1 Centre de Recherche des Cordeliers, INSERM, UMR S 872, 75006 Paris, France; 2 UPMC Univ Paris 06, UMR S 872, 75006 Paris, France; 3 Université Paris Descartes, UMR S 872, 75006 Paris, France; 4 «Inflammation, dégénérescence et remodelage vasculaire dans les pathologies rétiniennes», Institut de la Vision, INSERM UMRS 968, 17 rue Moreau, 75012 Paris, France; 5 Institut de la Vision, UPMC Univ Paris 06, UMR_S 968, 75012 Paris, France; 6 CNRS, UMR_7210, 75012 Paris, France; 7 CIRB, Collège de France, CNRS UMR 7241/Inserm U1050, Paris, France; 8 Sisène; Paris Biotech Santé, Paris, France; 9 CEA Grenoble, DSV/iRTSV/BGE(INSERM U-1038)/Biomics, 17 rue des Martyrs, 38054 Grenoble, France; 10 Plateforme d’imagerie, Centre de Recherche des Cordeliers, Université Pierre et Marie Curie, UMR S 872, Paris, France; 11 Assistance Publique des Hôpitaux de St Etienne, Centre hospitalier universitaire de Saint-Etienne, Université Jean Monnet; Faculté de Médecine, St Etienne, France; 12 CIISA, Faculdade de Medicina Veterinária, Universidade Técnica de Lisboa, Avenida da Universidade Técnica, 1300-477 Lisbon, Portugal; 13 Instituto Gulbenkian de Ciência, 2781-901 Oeiras, Portugal; 14 Hôtel-Dieu Hospital, Department of Ophthalmology, APHP, Paris, France; 15 Equipe 14, Institut de la Vision, 17, rue Moreau, 75012 Paris, France

**Keywords:** Angiogenesis, Notch, DLL4, Eye, Age related macular degeneration, Macrophages

## Abstract

Inflammatory neovascularization, such as choroidal neovascularization (CNV), occur in the presence of Notch expressing macrophages. DLL4s anti-angiogenic effect on endothelial cells (EC) has been widely recognized, but its influence on Notch signaling on macrophages and its overall effect in inflammatory neovascularization is not well understood. We identified macrophages and ECs as the main Notch 1 and Notch 4 expressing cells in CNV. A soluble fraction spanning Ser28-Pro525 of the murine extracellular DLL4 domain (sDLL4/28-525) activated the Notch pathway, as it induces Notch target genes in macrophages and ECs and inhibited EC proliferation and vascular sprouting in aortic rings. In contrast, sDLL4/28-525 increased pro-angiogenic VEGF, and IL-1β expression in macrophages responsible for increased vascular sprouting observed in aortic rings incubated in conditioned media from sDLL4/28-525 stimulated macrophages. In vivo, Dll4^+/−^ mice developed significantly more CNV and sDLL4/28-525 injections inhibited CNV in Dll4^+/−^ CD1 mice. Similarly, sDLL4/28-525 inhibited CNV in C57Bl6 and its effect was reversed by a γ-secretase inhibitor that blocks Notch signaling. The inhibition occurred despite increased VEGF, IL-1β expression in infiltrating inflammatory macrophages in sDLL4/28-525 treated mice and might be due to direct inhibition of EC proliferation in laser-induced CNV as demonstrated by EdU labelling in vivo. In conclusion, Notch activation on macrophages and ECs leads to opposing effects in inflammatory neovascularization in situations such as CNV.

**Electronic supplementary material:**

The online version of this article (doi:10.1007/s10456-012-9290-0) contains supplementary material, which is available to authorized users.

## Introduction

Angiogenesis is a hallmark in development, tissue repair and in pathological conditions such as cancer and age related macular degeneration (AMD) [1]. In angiogenesis, specialized endothelial cells at the tip of the forming vascular sprout (“tip cells”) guide the proliferating, lumen forming, “stalk cells” [2] to form new blood vessels. Delta-like 4 (DLL4) has been shown to play an important role in this process.

DLL4 is a member of membrane-bound proteins (Delta-like 1, 3, and 4 and Jagged1 and 2). They can activate four different Notch receptors (Notch1–4) [3]. Ligand binding to Notch induces the proteolytic release by the γ-secretase of the Notch intracellular domain (NICD) that translocates to the nucleus where it regulates gene expression of specific target genes such as HES1 and HEY1 [4]. Soluble Notch ligands can act as agonists [5–7] or antagonists [8, 9]. To activate Notch, it has been proposed that the Notch ligand has to be fixed to a support to enable it to tether the Notch extracellular domain away from the NICD (for review see D’Souza [4]). The report of soluble peptide agonists questions this hypothesis [6].

Notch-signaling pathways are involved in lateral inhibition in the differentiation of a variety of tissues [3]. Tip cells and arterial endothelial cells specifically express DLL4 [10, 11]. DLL4 down regulates VEGF receptor (VEGFR) expression on neighbouring stalk cells and inhibits their differentiation into tip cells and their proliferation [12–15]. It is regulated by VEGF and has been proposed to act as a negative feedback to prevent over exuberant angiogenic sprouting and to allow the formation of an orderly vascular network [14, 16]. DLL4 exerts this effect via Notch 1 and Notch 4 activation [10, 17], the two DLL4 receptors expressed on endothelial cells [11], but does not bind to Notch 3 [18]. DLL4-mediated Notch signaling is essential for embryonic vascular development [16, 19, 20]. Pharmacological DLL4 inhibition leads to exuberant but malfunctioning vessel formation in tumors and consequently tumor growth inhibition [21] and has been proposed as a possible therapy of tumors [21, 22]. Recent evidence that chronic DLL4 inhibition induces vascular neoplasms [23] casts doubt on the safety of this approach.

In neovascularization, observed in ischemia, tumor growth and inflammatory conditions, infiltrating cells from the myeloid lineage, such as macrophages, are key players [24–26] as an abundant source of angiogenic factors [1, 24, 27], notably vascular endothelial growth factor (VEGF), interleukin 1β, TNF-α, and interleukin 6 [27]. Notch signaling plays an important role in myeloid cell differentiation [28] and in the regulation of cytokine expression in mature macrophages [29–31]. Resting macrophages express all four Notch receptors [29, 30] and activated macrophages selectively further increase Notch 1 expression [30]. Notch pathway inhibition in bone marrow derived macrophages leads to an M2 (angiogenic) polarization of macrophages [31, 32]. Myeloid specific Notch-1 deletion decreases monocytic VEGFR1 expression and monocyte recruitment to the injury site [31]. Furthermore, systemic soluble DLL4 over-expression enhances leukocyte recruitment and leads to chaotic neovascularization in ischemia [9]. The influence of DLL4 and Notch signaling on macrophage angiogenic factor release is unknown. Taken together Notch signaling in monocyte recruitment and macrophage activation in pathological neovascularization is still ill defined.

Age-related Macular Degeneration (AMD) is the leading cause of irreversible blindness in the world [33]. The fast progressive “wet” form is characterized by leaky subretinal choroidal neovascularisation (CNV) that leads to extravasations and hemorrhage into the photoreceptor cell layer and photoreceptor cell loss [33]. CNV are dependent on vascular endothelial growth factor release [34] from the retinal pigment epithelium and macrophages [35, 36] that accumulate in the subretinal space in AMD [37]. The recruitment of monocytes to the subretinal space and the release of VEGF have been shown to be crucial in the development of “wet” AMD [38, 39].

We here investigated the role of DLL4 signaling in macrophages and endothelial cells and its overall effect on CNV.

## Materials and methods

### Animals

Twelve-week old C57Bl/6J mice (Janvier, France) and 6-month old *Dll4* ^+/−^ mice bred on a CD1 background [20, 40] and CD1 (*Dll4* ^+/+^) control mice (Janvier, France) were used. Mice were kept in specific pathogen-free conditions with food and water available ad libitum and housed in a 12/12 h light/dark cycle. The care and use of the animals was in compliance with the Centre de Recherche Committee for animal experimentation.

### CNV induction

Mice were anesthetized by peritoneal injection of ketamine (50 mg/kg) and xylazine (10 mg/kg). In pigmented animals (C57BL6) the pupils were fully dilated with 1 % tropicamide. Cover slips positioned on the mouse cornea were used as a contact glass. Laser-coagulations were performed 4–5 disc diameters away from the papillae with an Argon laser (532 nm, 400 mW, 50 ms and 50 μm). Treatment consisted either of 4 μg of recombinant soluble DLL4 (R&D systems Europe, France) diluted in 2 μl or vehicle, injected in the vitreous at 0 and 4 days of laser injury.

In albino CD1 (*Dll4* ^+/+^) and *Dll4* ^+/−^ mice we induced CNV by “cryogenic lesion”, as laser-induced RPE lesions cannot be induced with a 532 nm laser in albino animals. Briefly, a RPE/Bruchs membrane lesion was produced by inserting a liquid nitrogen cooled 30 gauge syringe through the sclera. The syringe was left in place for 5 s inducing the death of RPE cells. This injury results in CNV similar to the laser-induced model in pigmented animals.

After sacrifice the eyes were fixed in PBS/PAF 4 %. The eyes were either cryo-sectioned for immunohistochemistry of Notch receptors at 3 days or the choroids were dissected and CNV was quantified on CD102 stained flat mounts at 14 days using Image J Software (NIH, USA).

For determination of ECs proliferation in the laser impact in vivo, mice were injected i.p. daily with EdU (5-ethynyl-2′-deoxyuridine; 50 mg/kg body weight). Choroid whole mounts collected at day 4 following CNV induction were stained with collagen IV and the Click-iT® EdU A-594 Imaging Kit following manufacturer’s instructions (Invitrogen, France).

### Immunohistochemistry

Retinal and choroidal human whole mounts were post-fixed with cold acetone for 15 min. After washing with PBS, whole mounts were incubated overnight with primary antibodies diluted in PBS Triton 0.1 %. Mouse eye sections were incubated in PBS diluted antibodies. The following primary antibodies were used: human/mouse Notch1 (AF3647, R&D systems, France), rabbit polyclonal anti human/mouse Notch4 (ab23427, Abcam, Cambridge, UK), rabbit anti human/mouse HES1 (ab71-559, Abcam, Cambridge, UK), rabbit anti human DLL4 (ab7280, Abcam, Cambridge, UK), rat anti-mouse CD102 (clone 3C4, BD Biosciences Pharmingen), rat anti-CD31 (Clone MEC 13.3, BD Biosciences Pharmingen), rabbit polyclonal antibody anti IBA1 (Wako pure chemical industries; Osaka, Japan), rabbit anti VEGF (sc-507, Santa Cruz), rat anti mouse CD68 (MCA1957S, Abd Serotec, UK), rat anti-F4/80 (MCA497G, Abd Serotec, UK) and goat anti human collagen IV (Abd Serotec, UK). 1:400 PBS diluted corresponding Alexa-fluorescent-conjugated secondary antibodies (Invitrogen, OR, USA) were used to reveal the primary antibodies. Sections and whole mounts were viewed with a fluorescence microscope (DM5500 B Leica, France) or a confocal microscope (Zeiss LSM 510 or LSM 710). All immuno-staining were repeated at least three times. Staining that omitted the primary antibody served as negative controls.

### RNA Isolation and quantitative reverse transcription PCR

Total RNA were extracted with NucleoSpin RNAII kit (Macherey–Nagel EURL, France) and transcribed into cDNA. The cDNA product was amplified using Power SYBR Green PCR Master Mix (Applied Biosystems, France) and a Light Cycler 1.5 apparatus. The primers used for PCR are presented in Table 1. The mRNA levels were normalized to β actin mRNA.

**Table 1:** Primers used for quantitative PCR

Genes	Forward primers	Reverse primers
mACTIN	AAGGCCAACCGTGAAAAGAT	GTGGTACGACCAGAGGCATAC
mHESl	ACACCGGACAAACCAAAGAC	CGCCTCTTCTCCATGATAGG
mIL-lβ	CATGGAATCCGTGTCTTCCT	GAGCTGTCTGCTCATTCACG
mlL-6	GTGGCTAAGGACCAAGACCA	ACCACAGTGAGGAATGTCCA
mTNF-α	GCTTTCGGAACTCACTGGAT	ACATCTTCAGCAGCCTTGTG
mVEGF	GTGAGCCAGGCTGCAGGAAG	GAATGCGTCTGCCGGAGTCT
hACTIN	CCCAGCACAATGAAGATCAA	CGATCCACACGGAGTACTTG
hHESl	AGTGAAGCACCTCCGGAAC	CGTTCATGCACTCGCTGA
hHEYl	C6AGCTGGAC6AGACCAT	GGAACCTAGAGCCGAACTCA

### Enzyme-linked immune-adsorbent assay (ELISA)

Mouse VEGF concentrations were measured in lysates of retina and choroid, and in macrophage supernatants using ELISA assay following manufacturer’s protocol (R&D System, France).

### Peritoneal macrophages preparation and stimulation

Mouse peritoneal exudate cells (PECs) were elicited by i.p. injection of 2 ml 3 % thioglycollate (T9032, Sigma) into 8–10-week-old C57BL/6j mice. After 4 days, PECs were isolated by flushing of the peritoneum with 4 ml ice-cold PBS containing 5 mM EDTA (Sigma). On one side PECs were incubated 6 h at 37 °C in a 5 % CO_2_ atmosphere in RPMI 1640 medium containing 10 % FCS in order to let macrophages adhere to the wells. The medium and floating cells were removed and replaced by RPMI 1640 without serum with or without 1 μg/ml of recombinant sDLL4/28-525. Supernatants were collected at indicated times for ELISA and macrophages were lysed in 300 μl RA1 buffer (Macherey–Nagel EURL, France) for mRNA extraction.

On the other side, four hundred thousand PECs per well were plated on 24 wells plate. PECs were cultivated 6 h in DMEM medium containing 10 % FCS in order to let macrophages adhere to the wells. The medium and floating cells were removed and replaced overnight by DMEM without serum and with or without 1 μg/ml of recombinant soluble DLL4. Macrophages were activated 24 h in DMEM without serum with LPS (0.1 μg/mL) and stimulated with sDLL4/28-525 (1 μg/mL) and DAPT (5 mg/mL). Supernatants were collected and pooled (n = 4) for aortic ring assay stimulation.

### Flow cytometry

One million mouse PECs were incubated in ice-cold PBS medium (Blocking buffer) containing 5 mM EDTA (Sigma Aldrich, France), 1 % FCS, 3 % normal rat and mouse serum, and 10 % mouse Seroblock (anti CD16/CD32, Abd Serotec, UK). Macrophages were stained 25 min on ice with APC-conjugated rat anti-mouse F4/80 (MCA497APC) and PE-coupled rat anti-mouse CD11b (MCA711PE, Serotec). Cells were washed and incubated in permeabilization buffer (eBiosciences) before staining with the Notch1 (AF3647, R&D systems, France) and Notch4 (ab23427, Abcam, Cambridge, UK) intracellular domain antibodies. After a second wash cells were incubated with FITC-labeled anti-sheep and rabbit antibodies, respectively. Acquisitions were performed on a LSRII cytometer (BD Bioscience). Expression of Notch1 and 4 by macrophages gated for CD11b-PE and F4/80-APC expression was analyzed and illustrated using the Flow Jo V7.9 software.

### CNV-recruited macrophages sorted by flow cytometry

Neovascularization was induced by ten laser impacts per eye in 11-week-old males C57Bl/6 J mice. Three days later, a single injection was done in the vitreous with 2ul PBS or recombinant soluble DLL4 (1ug/μl). The next day, eight control and eight DLL4-treated eyes were dissected to isolate the choroids. Tissues were dissociated in HBSS buffer containing Liberase LT (1,7 *Wünsch* unit/ml) and DNaseI (100ug/ml, Roche Diagnostics) 30 min at 37 °C. Cells were washed with ice-old HBSS then PBS. Whole cells were resuspended in Blocking Buffer 15 min at 4 °C. Then they were stained with APC-conjugated rat anti-mouse F4/80, PE-coupled rat anti-mouse CD11b and FITC-coupled rat anti-mouse LY6B.2 alloantigen (Clone 7/4, Abd Serotec). Recruited inflammatory macrophages were sorted directly in Lysis Buffer as CD11b^high^ F4/80^+^ LY6B.2^pos^. mRNAs were extracted using the Kit Nucleospin RNA XS (Macherey–Nagel).

### Western blot

PECs incubated with PBS or sDLL4/28-525 were lysed in ice-cold Tris–HCl 50 mM pH 6,8 and SDS 2 % and PMSF 2 mM and anti-protease cocktail (Sigma). Protein preparation, electrophoresis and transfer on nitrocellulose membrane were performed following supplier’s instructions (Invitrogen, France). The Notch4 intracellular domain (N4-ICD, 52Kda) was detected with the rabbit polyclonal anti human/mouse Notch4 (1:500, ab23427, Abcam, UK). Anti-β-actin (1:5000, Santa Cruz, US) was used to control for protein loading. Proteins were revealed by corresponding secondary horseradish peroxydase-conjugated antibodies (Vector, US).

### Aortic ring assay

Aortae from adult C57BL6 were cut into 1-mm-thick rings and covered with 20 μL of Matrigel (BD Biosciences, France). Aortic rings were cultured for 3 days in Dulbecco’s Modified Eagle’s Medium (DMEM, Invitrogen, France) containing 10 % FCS, 1 % penicillin/streptomycin, and 0.2 % fungizone. Explants were exposed to sDLL4/28-525 (2 μg/mL; R&D system, France), or supernatants harvested from LPS (0.1 μg/ml) activated macrophages under different conditions: (1) control; (2) sDLL4/28-525 (1 μg/mL) stimulated; (3) sDLL4/28-525 (1 μg/mL) and DAPT (5 mg/mL, Sigma Aldrich, France) stimulated; (4) sDLL4/28-525 (1 μg/mL) stimulated added to sVEGFR1 (0,15 mg/mL, R&D, France) and IL-1Ra (10 mg/mL, Biovitrum, Sweden), from day 3 to day 5 of culture. The surface covered by the aortic ring and the vascular sprouts was measured daily. Vascular sprouting was calculated for each day and aortic ring as the percentage of the area covered at day 3.

### Endothelial cell culture and proliferation assays in vitro

Human umbilical vein endothelial cells (HUVEC) were isolated by collagenase dissociation (Roche Diagnostic, France). HUVECs from 5 different donors were used between passage 4 and 6. Cells were cultured in EBM (Clonetics, France) supplemented with 10 % FCS and 2 ng/ml of VEGF every other day. The medium was changed every 4 days. Hes-1 and Hey-1 expression were analysed in control or sDLL4/28-525 (1 μg/ml) or sDL/28-525 and DAPT (5 mg/mL) incubated ECs by RT-PCR. For ^3^H-thymidine incorporation proliferation assay, two thousand cells per well were plated on a 96 well plate for 24 h in EBM medium containing 10 % FCS at 37 °C in a 5 % CO_2_ atmosphere. The cells were “starved” for 48 h in EBM medium containing 1 % albumax. HUVEC were stimulated 96 h with 20 ng/ml of VEGF and with 1 μg/ml of sDLL4/28-525. Radioactive ^3^H-thymidine (1 μCi/well) was added to each well during the last 16 h. Cells were washed before reading of their radioactivity; a direct measure of proliferation was performed in a beta counter (TopCount NXT, Perkin Elmer, USA).

### Endothelial cells apoptosis analysis by TUNEL and Annexin V detection kit

Sixteen thousand HUVEC, per well were plated on a 12 wells plate. HUVEC were cultivated in EBM 1 % albumax (Gibco™ AlbuMAX, Invitrogen) alone or with VEGF 50 ng/ml or VEGF and 1 μg/ml of DLL4 for another 24 h. Terminal deoxynucleotidyl transferase dUTP nick end labeling (TUNEL) assay was performed to determine the number of apoptotic cells as described by manufacturer’s protocol (In Situ Cell Death Detection Kit, Roche Diagnostics). For flow cytometry, cells were stained with AnnexinV-FITC apoptosis detection kit and Propidium iodide staining solution (eBiosciences, Clinisciences France) to detect apoptotic- (AnnexinV-positive, PI-negative) and necrotic- (AnnexinV-negative, PI-positive) cells using a FACScan cytofluorimeter using PC lysis II software (Beckton Dickinson CO, Mountain view, CA, USA). The total percentage of dying cells was considered as the sum of percentage of apoptotic and necrotic cells.

### Statistical analysis

Graph Pad Prism 5 (GraphPad Software) was used for data analysis and graphic representation. Data are presented as mean ± standard deviation (SD). Statistical comparisons used unpaired two-sample *t* tests for RT-PCR results, Mann–Whitney *t* tests for CNV areas comparison, one way ANOVA followed by the Bonferroni multiple comparison test for HUVEC proliferation assay and two way ANOVA followed by the Bonferroni multiple comparison test for growth of aortic ring kinetics. *P* values less than 0.05 were considered statistically significant.

## Results

### Notch1 and 4 are expressed in endothelial cells and macrophages in choroidal neovascularization

**Fig. 1:**
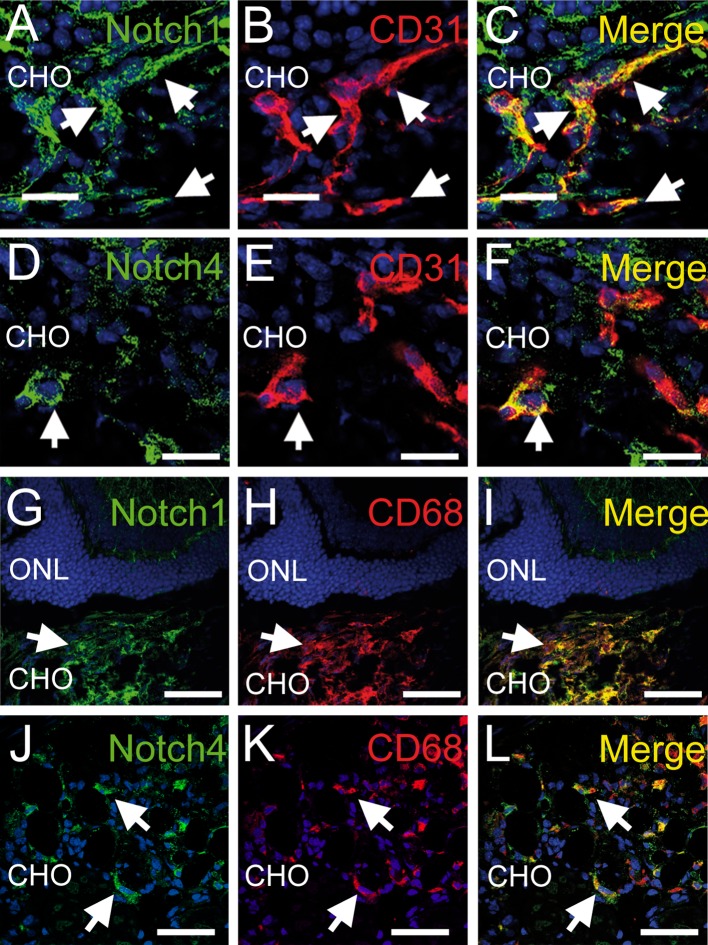
Notch1 and 4 expressions in mouse choroid during laser induced CNV. Confocal microscopy 3 days post laser induced CNV in mice: expression of Notch1 (*green*, **a**, **c**, **g** and **i**), endothelial cell marker CD31 (*red*, **b**, **c**, **e** and **f**), Notch 4 (*green*, **d**, **f**, **j** and **l**), macrophage marker CD68 (*red* **h**, **i**, **k** and **l**). Colocalization appears in *yellow*. Confocal microscopy optical section is 1.5 μm; *scale bars* represent 20 μm in **a**–**f** and 100 μm in **g**–**l**. All staining were performed 3 times with similar results. *ONL* outer nuclear layer; *CHO* choroid. (Color figure online)

DLL4 binds and activates Notch 1 and Notch 4 [10, 17] but does not bind to Notch 3 [18]. Notch 1 and 4 are expressed on retinal endothelial cells [11] during angiogenesis, but also on macrophages [29, 30] that participate in choroidal neovascularization (CNV) [38, 39]. To identify DLL4 sensitive cells in CNV we performed Notch 1 and Notch 4 immunohistochemistry on mice 3 days after CNV inducing laser-injury. Notch 1 staining (Fig. 1a) was detected in the CD31 positive (Fig. 1b, merge c) choroidal endothelial cells in CNV. Similarly, Notch 4 staining (Fig. 1d) was detected in the CD31 positive (Fig. 1e, merge f) choroidal endothelial cells in CNV. Choroidal vasculature in uninjured mice or distant from the laser-injury showed no staining (data not shown). Furthermore, 3 days after laser injury, Notch 1 staining (Fig. 1g) was detected in CD68 positive macrophages (Fig. 1h, merge i) adjacent to the laser-impact. To a lesser extent, Notch 4 (Fig. 1j) was detected in some, but not all, CD68 positive (Fig. 1k, merge l) cells. The photographs are representative of 3 independently performed experiments.

### sDLL4 activates Notch signaling and induces proangiogenic mediators in macrophages

**Fig. 2:**
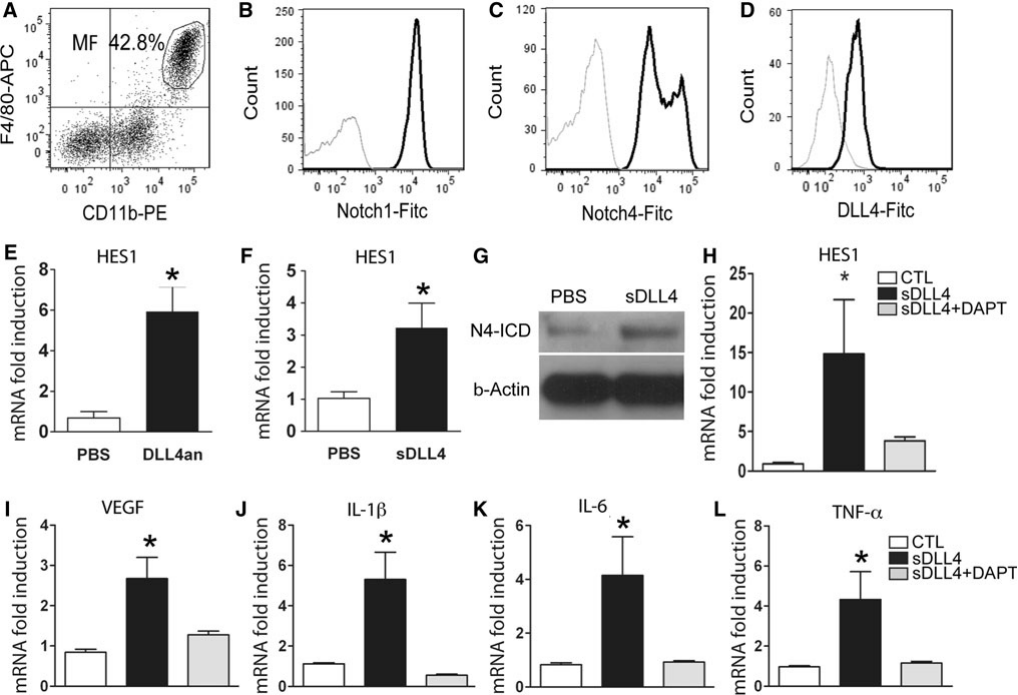
sDLL4 activates Notch signaling and induces proangiogenic mediators in macrophages in vitro: flow cytometry of F4/80^+^ CD11b^+^ macrophages (**a**), Notch1 (**b**), Notch4 (**c**) and DLL4 (**d**). HES-1 mRNA expression by PECs cultivated on sDLL4 coated plates (DLL4an = DLL4anchored) (**e**, PBS, *white*, n = 3, DLL4an, *black*, n = 3). HES-1 mRNA expression of soluble (s) DLL4 stimulated macrophages (**f**, PBS, *white*, n = 6 and sDLL4, *black*, n = 4). Western blotting of Notch-4 intracellular domain (N4-ICD) in macrophages stimulated with PBS or sDLL4 (**g**). mRNA expression in sDLL4 and sDLL4 and DAPT treated C57BL/6j activated macrophages of HES-1 (**h**), VEGF (**i**), IL-1β (**j**), IL-6 (**k**) and TNF-α (**l**). (LPS n = 4 *white*, LPS + sDLL4 n = 4 *black*, LPS + sDLL4 +DAPT n = 4 *grey*) * *p* < 0.05

Resting macrophages express all 4 Notch receptors [29, 30] and LPS stimulation selectively further increase Notch1 and DLL4 expression [30]. To analyze Notch 1 and Notch 4 expression on mouse peritoneal exudate cells (PECs), we gated on F4/80 and CD11b (Fig. 2a) and analyzed Notch 1 (Fig. 2b), Notch 4 (Fig. 2c) and DLL4 (Fig. 2d) expression. Anti-Notch 1 and anti-Notch 4 revealed with secondary FITC coupled antibodies both increased cell fluorescence of PECs prepared 4 days after thioglycollate injection.

Ligand binding to Notch induces the proteolytic release of the Notch intracellular domain (NICD) that translocates to the nucleus where it regulates gene expression of specific target genes such as HES1 and HEY1 [4]. To activate Notch, it has been proposed that the Notch ligand has to be fixed to a support to enable it to tether the Notch extracellular domain away from the NICD (for review see D’Souza [4]). However, recent evidence suggests that soluble Notch ligands can also act as agonists [5–7]. To test if the commercially available DLL4 (DLL4/28-525), composed of Ser28—Pro525 with a C-terminal 10-His tag, in its soluble or anchored form affects the Notch pathway in macrophages we incubated PECs from C57Bl6 mice with anchored DLL4/28-525 (Fig. 2e) or soluble DLL4/28-525 (Fig. 2f) and analyzed HES-1 induction (HEY-1 expression was undetectable in PECs, data not shown). Our results show that both, anchored and soluble DLL4/28-525 induced Notch downstream gene HES-1 similarly and significantly in macrophages. Furthermore, soluble DLL4/28-525 (sDLL4/28-525) increased Notch4 cleavage of the intra cellular domain (N4-ICD) (Fig. 2g) and inhibition of the γ-secretase by DAPT prevented the significant HES-1 mRNA induction of sDLL4/28-525 in activated macrophages in which an angiogenic state was induced by LPS incubation [41] (Fig. 2h). To evaluate if DLL4 induced Notch signaling influenced the expression of pro-angiogenic mediators, we incubated activated macrophages with sDLL4/28-525 and analyzed VEGF (Fig. 2i), IL-1β (Fig. 2j), IL-6 (Fig. 2k), and TNF-α (Fig. 2l) expression. sDLL4/28-525 significantly induced mRNA of all four genes and co-incubation with γ-secretase inhibitor DAPT prevented this effect.

### sDLL4 activates Notch signaling in HUVEC and inhibits their proliferation

**Fig. 3:**
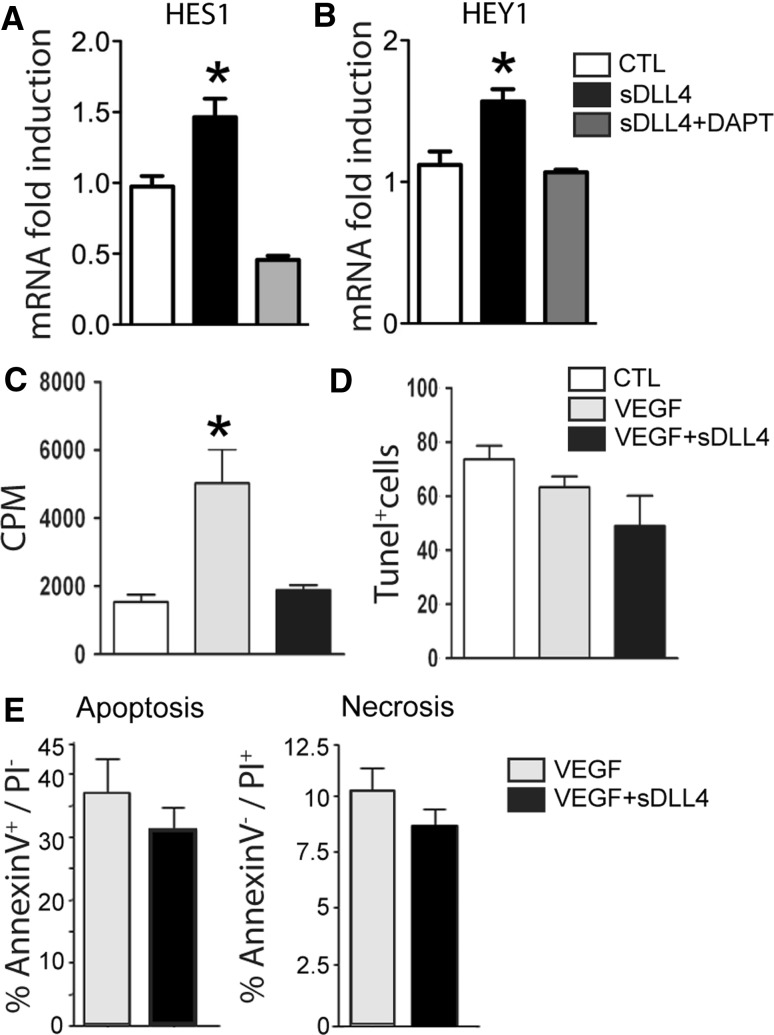
sDLL4 activates Notch in HUVECs and inhibits their proliferation: HES-1 (**a**, control (CTL), *white* n = 12; sDLL4, *black*, n = 12, sDLL4 + DAPT, *grey*, n = 6) and HEY-1 (**b**, CTL, *white*, n = 12; sDLL4, *black*, n = 12, sDLL4 + DAPT, *grey*, n = 6) mRNA expression by HUVEC stimulated with sDLL4 in vitro. Effect of sDLL4 on VEGF-induced HUVEC proliferation (**c**, CTL, *white*, n = 3, VEGF, *grey* n = 3, VEGF + sDLL4, *black*, n = 2) performed in triplicates. Percentage of TUNEL^+^ cells following starvation (**d**, CTL, *white*, n = 3, VEGF, *grey*, n = 3, VEGF + sDLL4, *black*, n = 2). Percent of apoptotic, or necrotic (**e**) HUVEC stimulated with VEGF (*grey*) or VEGF + sDLL4 (*black*) in minimum medium detected by annexin V—Propidium iodide staining by flow cytometry (n = 5). *CPM* counts per minute. * *p* < 0.05

To analyze if sDLL4/28-525 activates the Notch pathway in endothelial cells (EC), we incubated HUVEC with sDLL4/28-525 and analyzed HES-1 and HEY-1 expression. sDLL4/28-525 induced the Notch target genes HES1 and HEY1 significantly (although less strongly than in macrophages), γ-secretase inhibitor DAPT prevented the mRNAs induction (Fig. 3a, b). DLL4 induced Notch activation has been shown to inhibit EC proliferation [21]. To test the effect of sDLL4/28-525 on EC we exposed VEGF stimulated HUVEC to sDLL4/28-525 for 16 h and evaluated cell survival and proliferation. sDLL4/28-525 significantly reduced VEGF-induced HUVEC proliferation (Fig. 3c), as shown by ^3^H-thymidine uptake. No signs of sDLL4/28-525 induced cell death were detected by TUNEL assay (Fig. 3d) and no significant differences were found for apoptotic Annexin V positive—Propridium iodide (PI) negative HUVECs or necrotic Annexin V negative—PI positive HUVECs (Fig. 3e).

### sDLL4 directly inhibits vascular sprouting but accentuates the angiogenic phenotype of activated macrophages in vitro

**Fig. 4:**
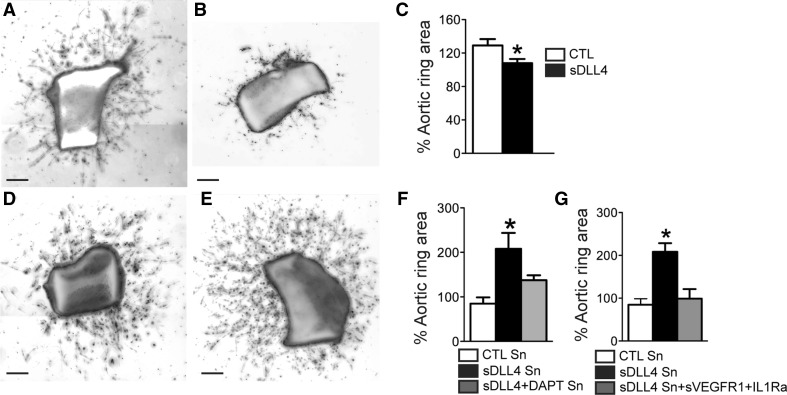
sDLL4 directly inhibits vascular sprouting but accentuates the angiogenic phenotype of activated macrophages in vitro. Photographs of aortic rings at day 5, incubated with PBS (**a**), or sDLL4 (**b**). **c** Areas of vascular sprouting in the presence of PBS (*white*, n = 16) or sDLL4 (*black*, n = 15) at day 5 and expressed as percentage of the aortic ring area at day 3, * *p* < 0.01. Photographs of aortic rings at day 5, incubated with conditioned medium of macrophages stimulated 24 h with LPS (**d**), or LPS + sDLL4 (**e**). **f**, **g** Quantification of areas of vascular sprouting at day 5 and expressed as percentage of the aortic ring area at day 3. **f** Effect of sDLL4 conditioned macrophage medium: supernatant of activated macrophages (*white*, n = 8); supernatant of sDLL4 stimulated activated macrophages (*black*, n = 8); supernatant of sDLL4 +DAPT stimulated activated macrophages (*grey*, n = 8)). **g** Effect of VEGF/Il-1β inhibition on sDLL4 conditioned macrophage medium: supernatant of activated macrophages (*white*, n = 8), supernatant of sDLL4 stimulated activated macrophages (*black*, n = 8); supernatant of sDLL4 stimulated activated macrophages supplemented with sVEGFR1 and IL-1Ra (*grey*, n = 8). *Sn* supernatant; * *p* < 0.001. *Scale bar* 50 μm

Activated macrophages are key players in pathological neovascularization [24–26] as an abundant source of angiogenic factors [1, 24, 27]. In neovascularization, therapeutically induced DLL4 signaling will affect EC proliferation but also potentially growth factor release from activated macrophages. To study the differential effect on ECs and macrophages we used the aortic ring assay. Aortic rings were grown over a period of 5 days either in control conditions (Fig. 4a) or in the presence of sDLL4/28-525 from day 3 (Fig. 4b). Quantification of vascular growth, expressed as the surface covered by endothelial sprouts relative to the pre-exposure surface at day 3, shows a small but significant inhibition of vascular sprouting by sDLL4/28-525 at day 5 (Fig. 4c). In a second set of experiments we exposed aortic rings from day 3 to day 5 to conditioned media from control activated macrophages (Fig. 4d) or from sDLL4/28-525 incubated activated macrophages (Fig. 4e). Interestingly, aortic rings exposed to supernatants from sDLL4/28-525-stimulated macrophages showed a significant increase in vascular sprouting compared to control supernatants which was reversed when sDLL4 stimulated macrophages were inhibited by γ-secretase inhibitor DAPT (Fig. 4f). We have shown that sDLL4/28-525-stimulated macrophages produce a variety of potential proangiogenic factors, notably VEGF and IL-1β (Fig. 2), two factors that have been shown to influence vascular outgrowth in aortic rings [42, 43]. To evaluate if the sDLL4-induced VEGF and IL-1β were responsible for the increased aortic ring sprouting, IL-1 receptor antagonist (IL-1Ra) and soluble VEGF receptor 1 (sVEGFR1) were added to the supernatants when administered to the aortic ring assay. Inhibition of VEGF and IL-1β completely reversed the proangiogenic effect of sDLL4/28-525-stimulated macrophage conditioned media (Fig. 4g).

### DLL4 mediated Notch activation inhibits choroidal neovascularization

**Fig. 5:**
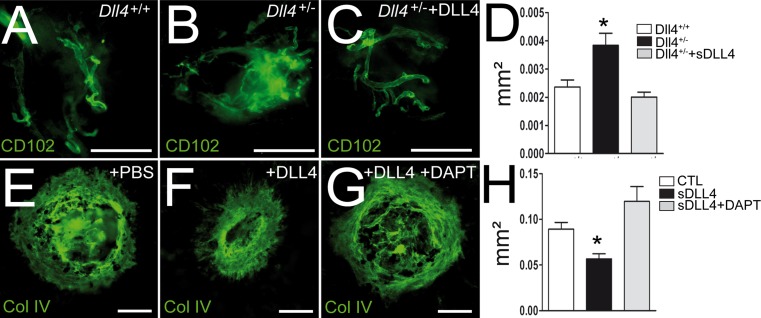
sDLL4 activates the Notch pathway in vivo and significantly inhibits choroidal neovascularization (*CNV*). Representative CD102-stained choroidal flatmount 14 days after cryo-injury induced CNV in *Dll4* ^+/+^ (**a**), *Dll4* ^+/−^ mice (**b**) and a *Dll4* ^+/−^ mice injected with sDLL4 (**c**). **d** Quantification of surface covered by CNV in *Dll4* ^+/+^ (*white*, n = 18 lesions), *Dll4* ^+/−^ (*black*, n = 19 lesions) and sDLL4 injected Dll4^+/−^ mice (*grey*, n = 7 lesions) * *p* < 0.05. Representative Collagen IV (Col IV)-stained choroidal flatmount 7 days after laser-induced CNV in C57BL/6 mice treated with PBS (**e**), sDLL4 (**f**) or sDLL4 and DAPT (**g**). **h** Quantification of surface covered by CNV in PBS treated mice (*white*, n = 35 impacts), sDLL4 treated mice (*black*, n = 30 impacts) and sDLL4 and DAPT treated mice (*grey*, n = 14 impacts) * *p* < 0.01. *Scale bar* 100 μm

Laser coagulation of the mouse choroid in pigmented C57Bl6 animals induces rupture of Bruchs membrane, a local inflammatory reaction and choroidal neovascularization (CNV) similar to “wet” AMD. *Dll4* ^+/−^ C57BL6 mice are not viable [20] and to dispose of adult *Dll4* ^+/−^ mice, we outbred the mice to a CD1 albino background. To induce CNV in albino mice we provoked a local injury using liquid nitrogen cooled needle that reproducibly induces CNV in pigmented as well as albino animals (complementary figure). CD102 stained choroidal flatmounts of cryo-injured *Dll4* ^+/+^ CD1 mice (Fig. 5a) were significantly smaller than in *Dll4* ^+/−^ CD1 mice (Fig. 5b). Furthermore, sDLL4/28-525 injection in *Dll4* ^+/−^ CD1 mice significantly reduced CNV development (Fig. 5c, CNV were quantified as CD102 positive surface on flatmounts Fig. 5d). PBS control injections in *Dll4* ^+/−^ CD1 mice had no effect (Data not shown). Similarly, sDLL4/28-525 injections significantly inhibited laser-induced CNV (Fig. 5f) in wild type C56BL6 mice compared to PBS injections (Fig. 5e). Disruption of DLL4 signaling by γ-secretase inhibitor DAPT completely reversed the anti-angiogenic effect of sDLL4/28-525 (Fig. 5g, CNV were quantified as collagen IV positive surface on flatmounts Fig. 5h).

### Local sDLL4 administration does not alter macrophage recruitment but increases macrophage VEGF expression in vivo

**Fig. 6:**
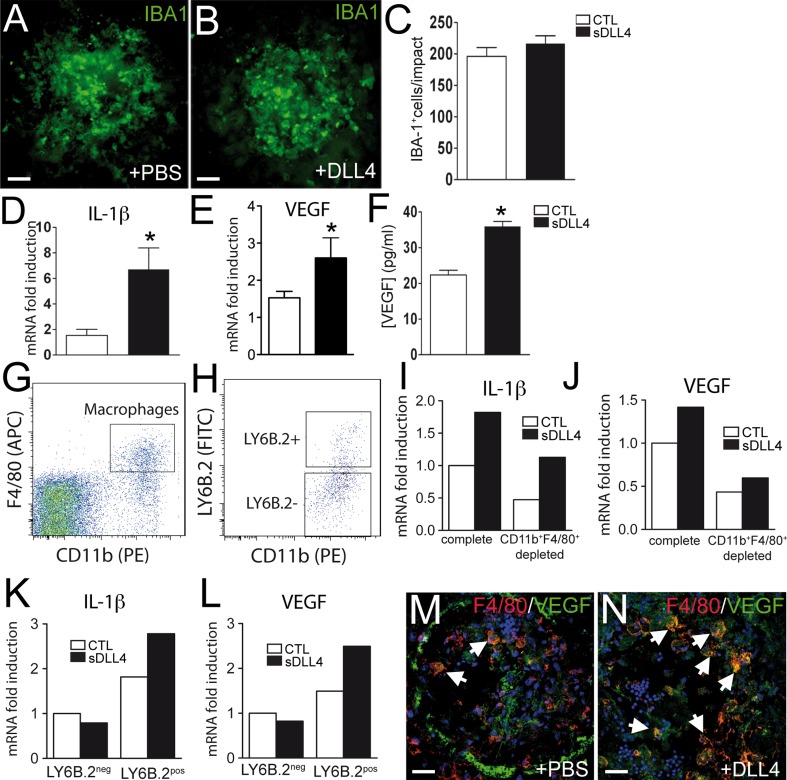
Influence of sDLL4 on macrophages in CNV in vivo. Representative image of an IBA1-stained choroidal flatmount 4 days after laser-injury of PBS treated mice (**a**) or sDLL4 treated mice (**b**). Quantification of IBA1 positive cells per impact in PBS (*white*, n = 25 impacts) and sDLL4 (*black*, n = 25 impacts) treated mice (**c**). Il-1β (**d**) and VEGF (**e**) mRNA levels and VEGF protein concentrations (**f**) in choroid 4 days following CNV and 24 h after PBS or sDLL4 injections (PBS, *white*, n = 6 choroid, sDLL4 *black*, n = 6 choroid). * *p* < 0.001. Fluorescent activated cell sorting (*FACS*) for CD11b and F4/80 (**g**) and LY6B.2 (**h**) on choroidal cell suspensions 4 days after laser-injury and 24 h after PBS (*white columns* **i**–**l**) or sDLL4 (*black columns* **i**–**l**) treatment. VEGF (**j**) and Il-1β (**i**) RT-PCR on sorted CD11b^+^F4/80^+^LY6B.2^neg^ resident macrophages and CD11b^+^F4/80^+^ LY6B.2^pos^ inflammatory macrophages. VEGF (**l**) and IL-1β (**k**) RT-PCR on whole choroidal cell suspension and CD11b^+^F4/80^+^depleted cell suspension. Choroidal cell suspensions were prepared from 8 choroids per group. The results are representative of two independent experiments. Double labeling of VEGF (*green*) and F4/80 (*red*) on choroidal flatmounts of PBS (**m**) and sDLL4 (**n**) treated CNV 4 days after laser-impact. Double labeling was performed on n = 3 eyes/group. *Arrows* indicate F4/80 positive cells. *Scale bar* 50 μm. (Color figure online)

Notch1 activation has been shown to regulate vascular endothelial growth factor receptor-1 in macrophages and myeloid specific Notch1 deletion decreases macrophage recruitment to skin lesions [31]. On the other hand systemic DLL4 inhibition has been suggested to increase leukocyte recruitment to the ischemic muscle [9]. To test whether local activation of notch signaling via intravitreal administration of sDLL4/28-525 altered the macrophage recruitment to the laser-injury site we stained choroidal flatmounts of PBS (Fig. 6a) or sDLL4/28-525 (Fig. 6b) injected eyes with the monocyte macrophage marker IBA-1. Quantification of IBA-1 positive cells at day 4, when macrophage recruitment is maximal, revealed no difference in IBA-1 positive cells in the two groups (Fig. 6c).

Notch signaling in macrophages has been shown to regulate cytokine expression [31, 32] and we observed a Notch dependent induction of VEGF, IL-1β in sDLL4/28-525-incubated macrophages in vitro that induces increased vascular sprouting in aortic rings (Figs. 2, 4). To evaluate if intravitreal sDLL4/28-525 altered the expression of these cytokines in vivo we analyzed IL-1β and VEGF mRNA expression by RT-PCR (Fig. 6d, e) and VEGF protein expression by ELISA (Fig. 6f) 24 h after PBS or sDLL4/28-525 injection at 4 days. Our results show that sDLL4/28-525 significantly induces IL-1β and VEGF in vivo. To more specifically analayze if sDLL4/28-525 induces VEGF and IL-1β in activated macrophages in the in vivo experiments, we performed RT-PCR on cells sorted by fluorescent activated cell sorted (FACS). Cell suspensions were prepared from choroids 4 days after laser-injury having received PBS or sDLL4 injections at day 3. Experiments were performed on 8 pooled choroids per group. We gated CD11b^+^F4/80^+^ macrophages (Fig. 6g) and further differentiated into CD11b^+^LY6B.2^pos^ inflammatory macrophages and CD11b^+^LY6B.2^neg^ resident macrophages (Fig. 6h). IL-1β and VEGF mRNA levels were lower when choroidal extracts are depleted of CD11b^+^F4/80^+^ macrophages, confirming that macrophages are a significant source of IL-1β and VEGF in the model (Fig. 6i, j respectively). While sDLL4 had little effect on CD11b^+^F4/80^+^ LY6B.2^neg^ resident macrophages, IL-1β and VEGF mRNA expression increased in the pooled CD11b^+^F4/80^+^ LY6B.2^pos^ inflammatory macrophages in the sDLL4 injected group (Fig. 6k, l respectively). Immunohistochemistry of VEGF (green) and the macrophage marker F4/80 (red) of PBS (Fig. 6m) and sDLL4/28-525 (Fig. 6n) injected eyes at 4 days reveals F4/80 positive macrophages in both groups (arrows). However, in sDLL4/28-525-injected eyes macrophages positive for VEGF (double labeling yellow, white arrows) were more numerous.

### sDLL4 inhibits endothelial cell proliferation in CNV in vivo

**Fig. 7:**
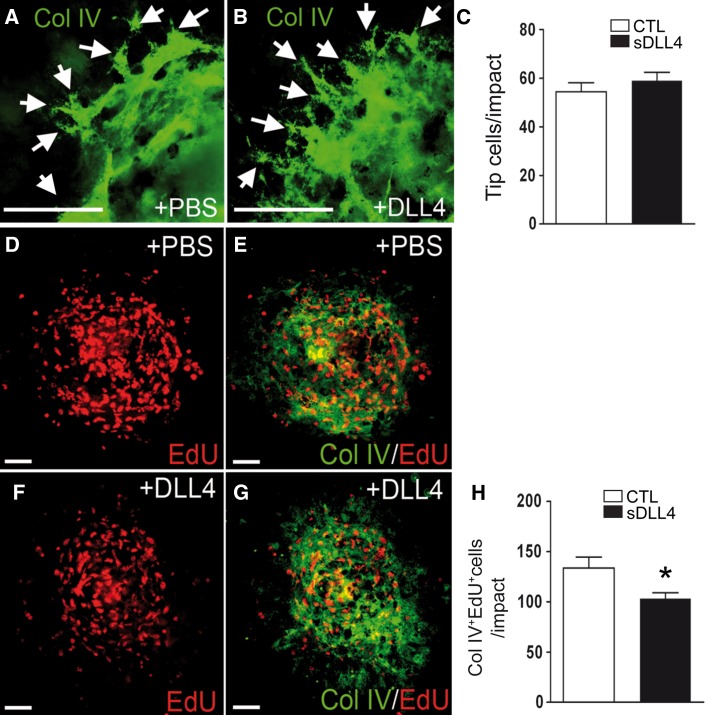
sDLL4 has no effect on Tip cells formation but inhibits endothelial cell proliferation in vivo. Representative image of tip cells (*arrows*) in a Collagen IV-stained choroidal flatmount 3 days after laser-injury in PBS (**a**) and sDLL4 (**b**) treated mice. Number of tip cells per impact (**c**, PBS, *white*, n = 22 impacts; sDLL4, *black* n = 23 impacts). Double labeling of EdU (**d**–**g**, *red*) and Collagen IV (**e**, **g**, *green*) on choroidal flatmounts of PBS (**d**, **e**) and sDLL4 (**f**, **g**) treated mice 4 days after laser-impact. Quantification of EdU^+^Collagen IV^+^ endothelial cells (**h**, PBS, *white*, n = 14 impacts; DLL4, *black*, n = 18 impacts). * *p* < 0.05, Col IV, Collagen IV. *Scale bar* **a**–**g** 50 μm. (Color figure online)

Dll4 heterozygosity or DLL4 inhibition using specific antibodies dramatically increases the number of tip cells and the density of the vascular plexus in retinal angiogenesis [12, 14, 21]. First, we screened Collagen IV stained choroidal flatmounts at different time points after laser-injury for the presence of tip-cells, which could be detected at day 3. To evaluate if sDLL4/28-525 changes the number of tip cells in CNV we counted the number of tip cells in Collagen IV stained choroidal flatmounts of PBS (Fig. 7a) and sDLL4/28-525 (Fig. 7b) treated mice 3 days after laser-injury. sDLL4/28-525 had no effect on tip-cell formation, as tip cell counts in both groups were similar (Fig. 7c). To study if sDLL4/28-525 changes the proliferation rate of endothelial cells in CNV in vivo, PBS treated (Fig. 7d, e) and sDLL4/28-525 treated (Fig. 7f, g) mice were injected with the artificial nucleotide EdU at day 3 and choroidal flatmounts were prepared at 24 h later and double labeled for EdU (red) and Collagen IV (green) to allow quantification of proliferating endothelial cells between 3 and 4 days. Quantification of Collagen IV EdU double-positive cells show a significant decrease in sDLL4/28-525 injected animals (Fig. 7h).

## Discussion

DLL4s anti-angiogenic effect on endothelial cells (EC) has been widely recognized, but its influence on Notch signaling on macrophages and its overall effect in inflammatory neovascularization is not well understood. We here show that Notch signaling on macrophages induces a pro-angiogenic phenotype in vitro and in vivo and possibly partly counterbalances the overall anti-angiogenic effect of Notch activation in inflammatory neovascularization.

DLL4 exerts its effect mainly via Notch 1 and Notch 4 [10, 17], while its affinity to Notch 3 is low [18]. To test the expression pattern of Notch 1 and Notch 4 in an inflammatory neovascularization we studied laser-induced CNV in mice eyes. In laser-induced CNV Notch 1 and Notch 4 expression was identified in macrophages, identified by CD68, but also in activated vascular endothelium, identified by CD31 positivity, in the vicinity of the laser burn (Fig. 1). We also confirmed the previously described expression of Notch 1 [29–31] but also Notch 4 on macrophages by flow cytometry on CD11b, F4/80 expressing thioglycolate elicited peritoneal macrophages (Fig. 2). Our results show that macrophages and activated endothelial cells are potentially DLL4 sensitive in inflammatory neovascularization such as CNV.

Soluble Notch ligands can act as agonists [5–7, 44] or antagonists [8, 9]. It has been postulated that Notch ligand extracellular domains have to be immobilized to activate Notch [4]. A soluble extracellular construct of DLL4 (amino acid residues 1–486 with a C-terminal His tag) has been shown to inhibit Notch signaling, evaluated by Notch target gene expression HES1 and HEY1 in vitro [18]. To influence Notch signaling we purchased the commercially available mouse soluble DLL4, composed of Ser28–Pro525 with a C-terminal 10-His tag. Surprisingly, the mouse sDLL4/28-525 induced the Notch target gene HES1 γ-secretase dependently in macrophages similarly to surface-fixed sDLL4/28-525 and it led to the cleavage of the Notch 4 intracellular domain in macrophages (Fig. 2). In HUVECs sDLL4/28-525 induced Notch target genes HES1 and HEY1 in a γ-secretase dependent fashion, although less potently compared to macrophages and might only partially activate the Notch pathway (Fig. 3). Nevertheless, it inhibited EC proliferation in vitro (Figs. 2, 3, 6) as previously shown for DLL4 dependent Notch activation [15, 21]. Moreover, it inhibited the exacerbated CNV of *Dll4* ^+/−^ CD1 mice and diminished CNV in C57BL6 mice in a Notch dependent fashion, as DAPT reversed its effect (Fig. 5). Taken together our data shows that the mouse sDLL4 Ser28–Pro525 activates Notch signaling in vitro and in vivo which is somewhat in contradiction with previous reports of a slightly longer human construct [18] and casts doubt on the paradigm that the Notch ligand extracellular domains have to be immobilized to activate Notch [4]. Similarly, amino acids 188–204 of the Notch ligand Jagged1 activates Notch in vitro and in vivo without immobilization [6, 7] suggesting that other factors determine the biological activity of Notch ligands.

DLL4 and Notch signaling have been shown to down regulate VEGF receptor (VEGFR) expression on stalk cells [12–14] and inhibit angiogenesis in vitro and in vivo [7, 9, 12–14, 16, 18–23]. Aortic rings prepared from Dll4^+/−^ mice develop significantly more vascular sprouts [12]. We here show that sDLL4/28-525 inhibited vascular sprouting significantly in the aortic ring assay (Fig. 4), which confirms that the murine sDLL4/28-525 activates the Notch pathway in vitro. To evaluate how DLL4 influences CNV in vivo, we compared CNV elicited by a cryo-injury in *Dll4* ^+/+^ CD1 and *Dll4* ^+/−^ CD1 mice. We show that *Dll4* ^+/−^ mice develop significantly more CNV than *Dll4* ^+/+^ control mice. sDLL4/28-525 injections, on the other hand, inhibited the exacerbated CNV *Dll4* ^+/−^ mice, suggesting that sDLL4/28-525 also acts as a DLL4 agonist in vivo (Fig. 5). Similarly, sDLL4/28-525 inhibited CNV and Notch signaling inhibitor reversed its effect in the classically used laser-induced CNV model of C57Bl6 mice. These findings underline the necessity to evaluate Notch activity of the different constructs and in different biological settings individually.

In neovascularization observed in ischemia, tumor growth and inflammatory conditions, infiltrating cells from the myeloid lineage (macrophages/microglial cells) are key players [24–26]. Notch pathway activation on bone marrow derived macrophages affects their cytokine expression profile [31, 32]. Although macrophages secrete a variety of pro- and anti-angiogenic mediators and no single factor determines their overall effect on neovascularization, we here show that sDLL4/28-525 induces VEGF, IL-1β, IL-6 and TNF-α in macrophages (Fig. 2), but also other pro-inflammatory factors such as CCL2 and IL-12 (data not shown). To test the net effect of macrophage Notch activation on their overall angiogenic potential, we grew aortic rings in culture medium from sDLL4/28-525 or control stimulated macrophages. Our data shows, that sDLL4/28-525 leads to a significant shift towards increased angiogenicity in macrophages (Fig. 4). This shift was Notch pathway dependent, as it was preventable when macrophages were co-incubated with the γ-secretase inhibitor DAPT. More specifically the pro-angiogenic effect of conditioned medium from sDLL4/28-525 stimulated activated macrophages depended on VEGF and IL-1β as their inhibition reversed the proangiogenic effect of the conditioned macrophage medium. We next evaluated its influence on macrophages (Fig. 5) in vivo. DLL4 inhibition has been shown to increase leucocyte recruitment and inhibit neovascularization in the ischemic hindlimb [9]. We show that leukocyte recruitment to the laser-induced injury is independent of local DLL4 signaling, as IBA-1 positive macrophage/microglial cell prevalence in PBS and sDLL4/28-525 at 4 days were comparable. We have shown that Notch activation induces VEGF, IL-1β, IL-6 and TNF-α in activated macrophages in vitro (Fig. 2) and increases the angiogenic potential in aortic rings in a VEGF/IL-1β dependent fashion (Fig. 4). Similarly, sDLL4/28-525 injections in vivo significantly increased intraocular IL-1β, VEGF mRNA and VEGF protein concentrations at 4 days, when macrophage recruitment is maximal [45]. Our data from RT-PCR performed on choroidal cell suspensions shows the important contribution of CD11b^+^F4/80^+^ macrophages in IL-1β and VEGF mRNA production and sDLL4/28-525 dependent induction in F4/80^+^CD11b^+^LY6B.2^pos^ inflammatory macrophages in vivo. VEGF immunohistochemistry at 4 days after laser injury confirms F4/80 positive activated macrophages as a major source of VEGF in sDLL4/28-525 injected eyes (Fig. 6). In view of Notch activating agents as therapeutics of inflammatory neovascularization, our results suggest that DLL4 signaling in activated macrophages in CNV in vivo can induce VEGF and possibly an angiogenic macrophage phenotype similar to that observed in vitro (Figs. 2, 4).

Despite the proangiogenic effect of sDLL4/28-525 injections on macrophages, DLL4 signaling significantly inhibits CNV (Fig. 4). This effect is possibly mediated by a direct effect on endothelial cells. DLL4 inhibition, by genetic or pharmacological means, prevents the differentiation of stalk cells into tip cells and their proliferation [12–14]. Our data shows, that Notch stimulation by sDLL4/28-525 injections did not increase the tip cell count, making this mechanism unlikely in our experimental setting. To evaluate the endothelial cell proliferation rate in CNV we systemically administered EdU in laser-injured mice at day 3 and evaluated EdU^+^CollagenIV^+^cells at day 4 (Fig. 7). EdU^+^CollagenIV^+^cells were significantly less numerous in sDLL4/28-525 eyes demonstrating a significant inhibition of endothelial cell proliferation over the relatively short time span of 24 h. DLL4 has been shown to inhibit EC proliferation in vitro and in vivo [12, 15, 21]. The suggestion that DLL4 regulates EC proliferation by VEGFR2 downregulation [15] has recently been challenged [46]. In our hands the observed sDLL4/28-525 induced inhibition of VEGF induced HUVEC proliferation was not accompanied by an alteration of VEGFR2 mRNA (data not shown) suggesting an alternative pathway.

Taken together, our results show that the soluble protein composed of the extracellular domain Ser28-Pro525 of murine DLL4 activates Notch as it induces Notch target genes, and it has the opposite effect of Dll4 heterozygosity in vitro and in vivo (vascular sprouting in aortic rings, and CNV). Our data shows that DLL4 signaling, although it accentuates the angiogenic potential of macrophages, inhibits neovascularization in an inflammatory setting possibly by a direct effect on endothelial cell proliferation. Our results suggest that inhibition of macrophage infiltration in situations of inflammatory neovascularization might increase the anti-angiogenic efficacy of DLL4 as a therapeutic agent.

## Electronic supplementary material

Below is the link to the electronic supplementary material.

Supplementary figure 1: Cryo-injury induced choroidal neovascularization: Choroidal flatmounts of C57BL/6j (A) and CD1 (B) in CD102 stained choroidal flatmounts 14d after liquid nitrogen cooled 30gauge syringe induced cryo injury. Supplementary material 1 (TIFF 2344 kb)
