# Supplementary figures and images for: Delta-like 4 inhibits choroidal neovascularization despite opposing effects on vascular endothelium and macrophages
Source: Angiogenesis. 2012 Aug 7;15(4):609–22. doi: 10.1007/s10456-012-9290-0 (PMC3496480; doi:10.1007/s10456-012-9290-0)

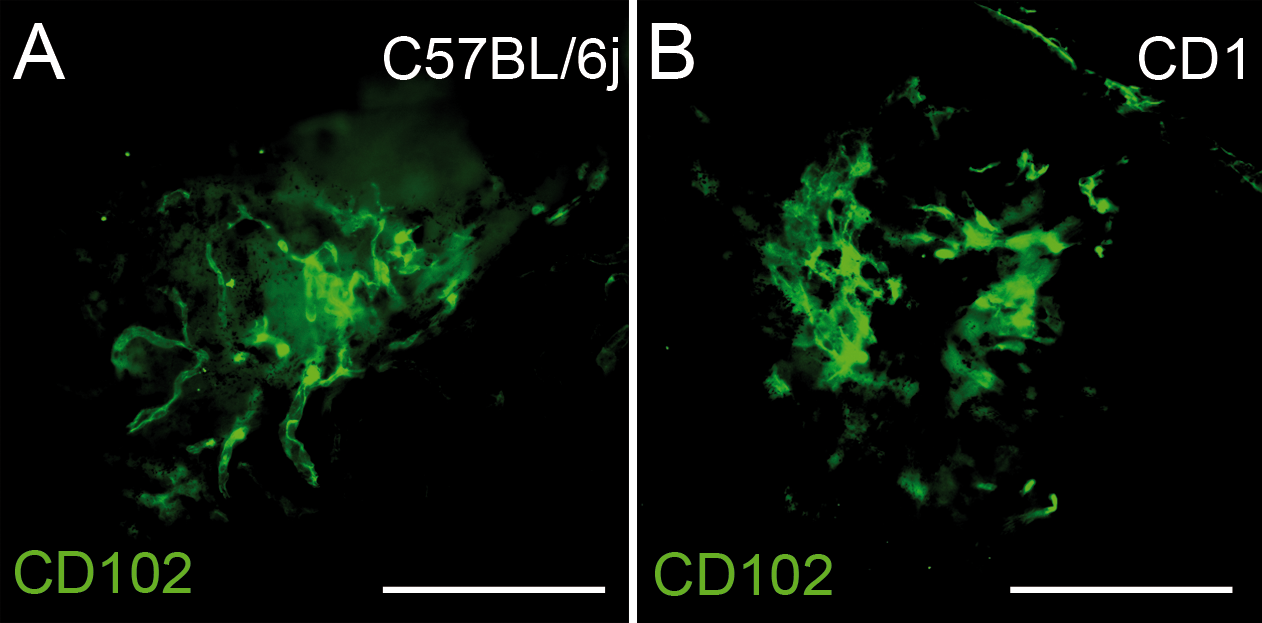

Supplement: Supplementary file 1 — Supplementary figure 1: Cryo-injury induced choroidal neovascularization: Choroidal flatmounts of C57BL/6j (A) and CD1 (B) in CD102 stained choroidal flatmounts 14d after liquid nitrogen cooled 30gauge syringe induced cryo injury. Supplementary material 1 (TIFF 2344 kb) [file 10456_2012_9290_MOESM1_ESM.tif]
